# Beyond Snoring: Unexpected Presentation of Obstructive Sleep Apnea

**DOI:** 10.7759/cureus.99866

**Published:** 2025-12-22

**Authors:** Pedro M Vieira, Ana Carolina Aranda, Filipe António C Martins, Inês A Ferreira, Margarida Barroso, Mariana Khomynets

**Affiliations:** 1 Family Medicine, Unidade Local de Saúde (ULS) da Lezíria, Santarém, PRT

**Keywords:** nail abnormalities, primary care, sleep apnea, sleep monitoring, young adults

## Abstract

Obstructive sleep apnea syndrome (OSAS) is a common yet often underdiagnosed sleep-related breathing disorder characterized by recurrent upper airway obstruction during sleep. We present a case of a 24-year-old, non-obese male patient with severe OSAS who exhibited an atypical clinical picture dominated by long-standing fatigue, poor concentration, and digital clubbing. Diagnostic evaluation included polysomnography, which confirmed severe OSAS, and ancillary testing to exclude pulmonary, cardiac, and gastrointestinal causes of clubbing. Continuous positive airway pressure (CPAP) therapy led to marked improvement in symptoms and daytime functioning. This case highlights the importance of early recognition of atypical OSAS presentations in primary care and reinforces the role of the family physician in coordinating diagnostic evaluation and treatment.

## Introduction

Obstructive sleep apnea syndrome (OSAS) is the most prevalent sleep-related breathing disorder [1]. It is characterized by repeated episodes of complete obstruction (causing apnea) or partial obstruction (causing hypopnea) of the upper airway during sleep, leading to fragmented sleep due to recurrent awakenings and possible hypoxia [2]. It is estimated that the prevalence of OSAS in the general population is 13% in men and 6% in women [3]. The pathophysiology results from the interaction between an unfavorable anatomy of the upper airway and sleep-induced physiological changes, such as decreased muscle activity, making the airway more collapsible [4].

The most common risk factors are obesity, older age, male gender, and anatomical characteristics of the upper airway, such as a large neck, narrow oropharynx with reduced space behind the uvula and soft palate, a high Mallampati score indicating reduced oropharyngeal space due to the relative size of the tongue base (typically class III or IV), enlarged palatine tonsils, bulky tongue base (macroglossia), retrognathism, or micrognathism [4]. OSAS is recognized as a risk factor for recurrent stroke and transient ischemic attacks [5]. In addition, increased severity of apnea is associated with higher all-cause mortality, especially in men under 50 years of age [6]. The most common symptoms include excessive daytime sleepiness, snoring, waking up feeling suffocated, and pauses in breathing during sleep [2].

In adults, the gold standard for diagnosing OSAS is polysomnography (PSG) performed in a laboratory, with an apnea-hypopnea index (AHI) ≥5 events per hour of sleep considered diagnostic. Correction of modifiable risk factors is recommended, including weight loss, changing sleeping position (in the case of positional OSAS), and avoiding alcohol consumption and the use of sedative medications [7-10]. Continuous positive airway pressure (CPAP) therapy is the standard treatment for adults with OSAS. It prevents respiratory events by maintaining positive transmural pressure in the pharynx, such that intraluminal pressure exceeds external pressure, and by increasing end-expiratory lung volume [11].

Conditions commonly associated with OSAS include pulmonary hypertension, cardiovascular and cerebrovascular events, metabolic syndrome, type 2 diabetes, hepatic steatosis, and neuropsychiatric disorders, while it can also be linked to adverse effects such as drowsiness while driving (with increased risk of traffic accidents) [12,13].

Questionnaires such as STOP-Bang, Epworth Scale, and Berlin Questionnaire should not be routinely used for OSAS screening in asymptomatic patients due to their poor validity [14]. However, they may be of some value in identifying OSAS in highly symptomatic and high-risk patients [15].

Digital clubbing - characterized by an increase in the mass of the distal tip of the finger and increased longitudinal and transverse curvature of the nail plate - is often associated with chronic hypoxia and several cardiopulmonary disorders, underscoring the need to explore OSAS as a potential contributing condition. It is the most common manifestation of hypertrophic osteoarthropathy, but it may also occur in association with numerous systemic conditions, including pulmonary diseases (such as lung cancer, interstitial lung disease and cystic fibrosis), cardiac disorders (notably cyanotic congenital heart disease and infective endocarditis), hepatic diseases (particularly cirrhosis and primary biliary cholangitis), and gastrointestinal conditions (including inflammatory bowel disease, celiac disease and gastrointestinal malignancies) [16,17]. The acquired bilateral form of digital clubbing is the most common presentation, typically beginning in the thumb and index finger [18-20]. This clinical case refers to a young adult whose longitudinal follow-up with the family physician allowed the identification of a common diagnosis with an atypical presentation, highlighting persistent complaints.

## Case presentation

This is a 24-year-old Caucasian male patient with a history of chronic rhinitis and ear, nose, and throat surgery in childhood (adenoidectomy, turbinectomy, and bilateral transtympanic tube placement). He is medicated with nasal corticosteroids, although with irregular use. He had no known drug allergies and denied smoking, alcohol use, or substance abuse. There was no relevant family history.

He consulted his family doctor (FD), reporting asthenia and difficulty concentrating, without any other associated symptoms at that time. According to his medical history, the complaints of asthenia had begun at approximately 13 years of age, although it initially did not interfere with his daily functioning. He performed well in school, slept approximately 8 h per night, maintained a healthy diet and lifestyle, and had no gastrointestinal tract changes. Later, when he started college, he developed complaints of difficulty concentrating and worsening asthenia, sometimes requiring an afternoon nap. 

On physical examination, he weighed 65 kg and was 177 cm tall (BMI: 20.75). He was in good general health, with well-colored, well-hydrated mucous membranes, and cardiopulmonary auscultation and abdominal examination revealed no apparent abnormalities. A summary analytical study was requested. The laboratory results showed hemoglobin within normal limits (13.8 g/dL), and iron deficiency based on low ferritin (29.1 ng/mL), with normal serum iron (iron 151 µg/dL). There were no changes in vitamin B12 levels, folic acid levels, or thyroid function (Table 1).

**Table 1:** Laboratory tests obtained during the diagnostic workup.

Blood tests	Value	Reference range
Hemoglobin	13.8 g/dL	13-16.5 g/dL
Leukocytes	6.6 × 10³/mm³	4.0-10.0 × 10³/mm³
Platelets	294 × 10³/mm³	140-440 × 10³/mm³
Glucose	77 mg/dL	50-99 mg/dL
Total cholesterol	178 mg/dL	<200 mg/dL
High-density lipoprotein (HDL) cholesterol	60 mg/dL	>45 mg/dL
Triglycerides	61 mg/dL	35-160 mg/dL
Uric acid	5.3 mg/dL	3.0-8.2 mg/dL
Creatinine	1.0 mg/dL	0.6-1.2 mg/dL
Ferritin	29.1 ng/mL	50-300 ng/mL
Iron	151 μg/dL	50-170 μg/dL
Total iron-binding capacity	467 μg/dL	250-450 μg/dL
Transferrin	281 mg/dL	200-400 mg/dL
Folic acid	5.2 ng/mL	>4.5 ng/mL
Vitamin B12	473 pg/mL	>200 pg/mL
Thyroid-stimulating hormone (TSH)	1.15 mIU/L	0.40-4.2 mIU/L
Thyroglobulin antibodies (TgAb)	Negative	-
Anti-thyroid peroxidase (anti-TPO) antibodies	Negative	-

Treatment with folic acid and iron (folic acid 1 mg and iron 90 mg, once daily on an empty stomach) was initiated for six months. A follow-up appointment was scheduled at the end of treatment; however, the patient did not attend.

**Figure 1:**
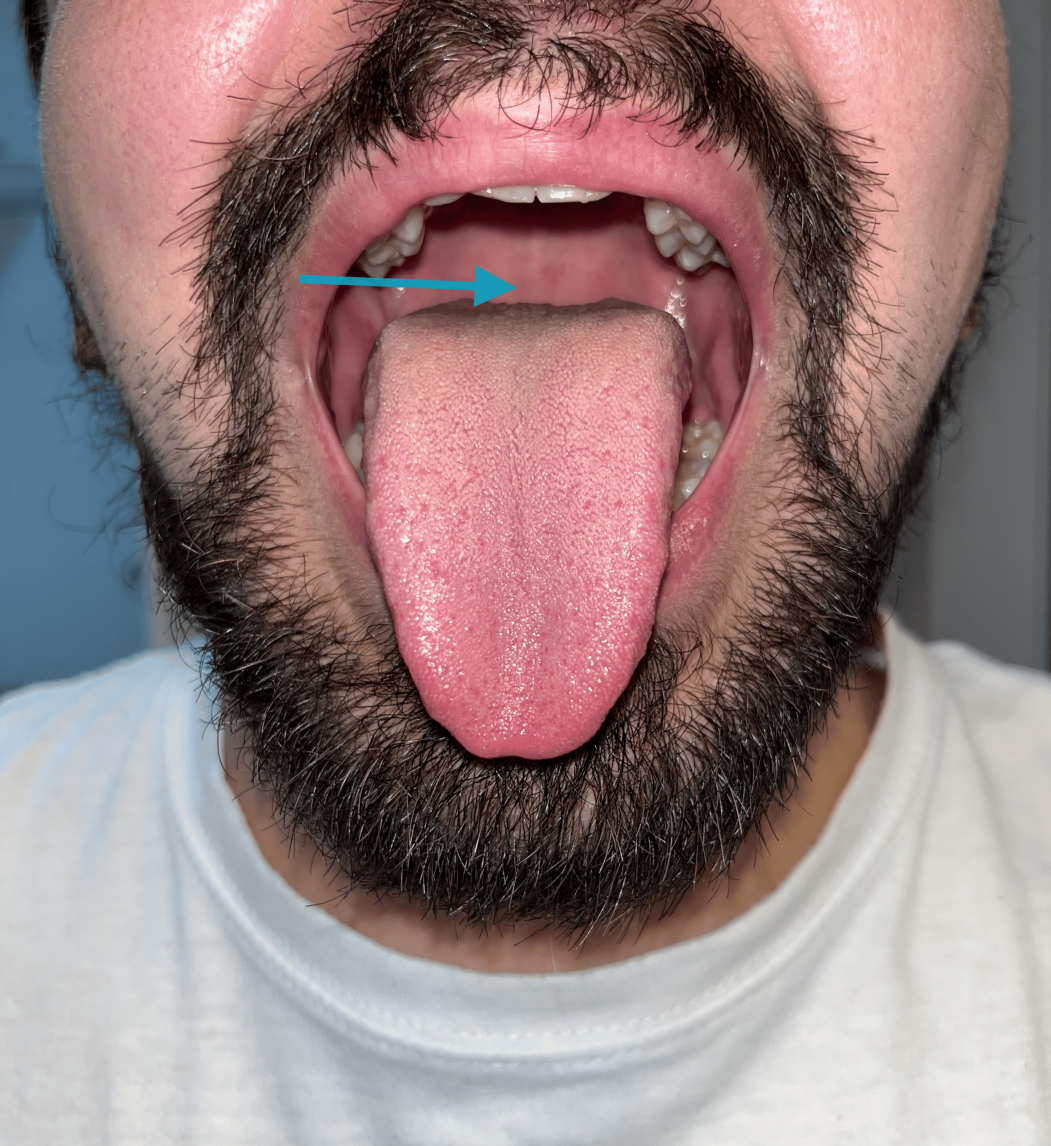
Inspection of the patient’s oropharynx. Mallampati class III score. Blue arrow: the soft palate is only partially visible, and the uvula is not visualized.

**Figure 2:**
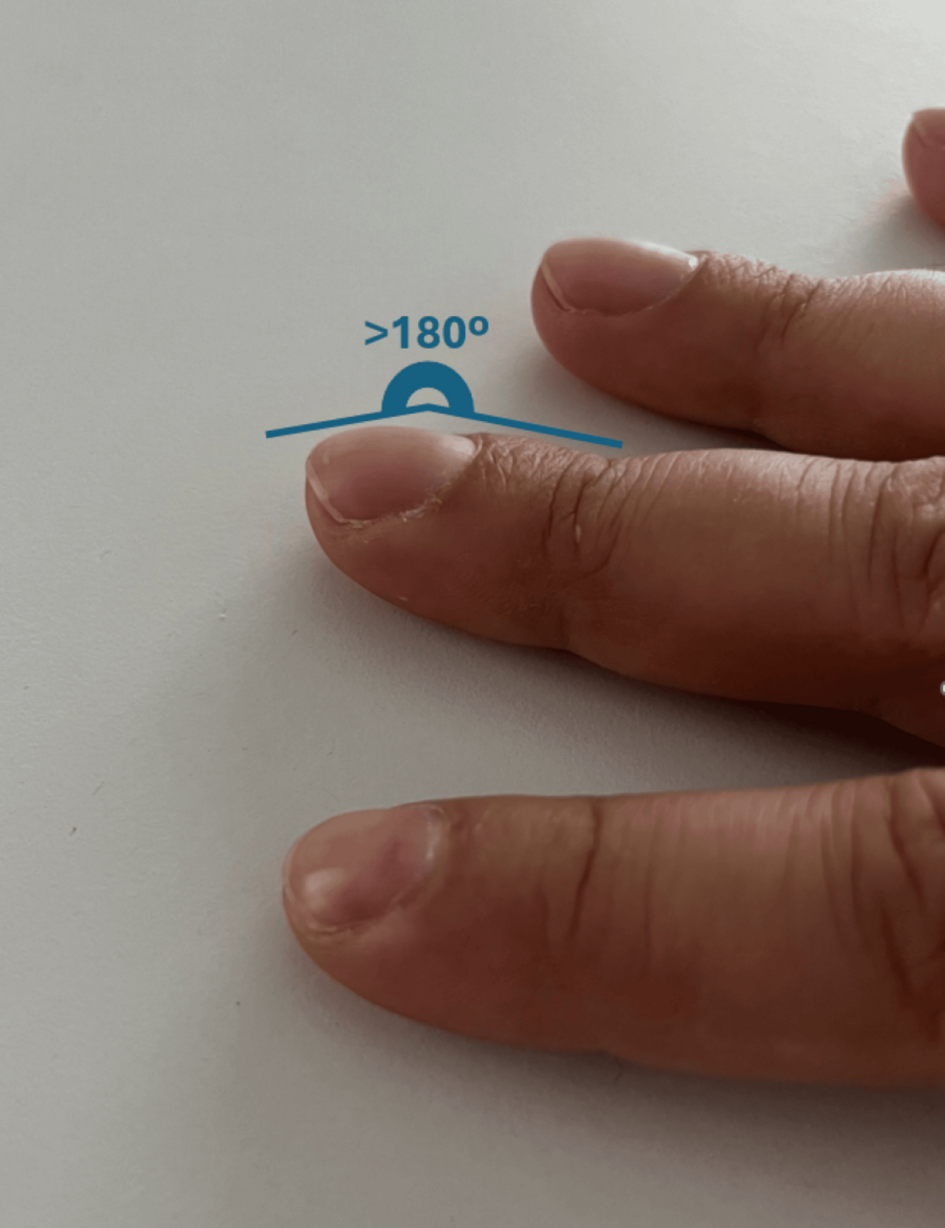
Digital clubbing: view of the patient’s fingers showing evident nail clubbing. Digital clubbing is multifactorial and appears to be related to chronic or repetitive hypoxemic exposure. The differential workup in this patient was negative for the most frequent etiologies, raising the possibility that chronic nocturnal hypoxemia from an early age may have played a contributory role.

Five years later, at the age of 29 years, the patient returned to the FD, reporting persistent asthenia, difficulty concentrating, and memory problems, revealing that the previous supplementation with folic acid and iron did not improve his complaints. He reported that during a vacation with friends, he was alerted to severe snoring and possible nocturnal pauses in breathing. He maintained his healthy lifestyle but increased his daily coffee consumption to six cups, with no perceived impact on his sleep. The physical examination during this appointment revealed Mallampati class III, digital clubbing, an Epworth Sleepiness Scale (ESS) score of 13 points (moderate daytime sleepiness), and a STOP-Bang score of 4 points (moderate risk of OSAS) (Figures 1, 2). Taking this into account, a level II polysomnography (PSG), spirometry, pulmonary computed tomography (CT scan), and echocardiogram (for differential diagnosis of pulmonary and extrapulmonary causes of clubbing) were requested.

The PSG confirmed severe obstructive apnea/hypopnea, with an overall apnea-hypopnea index (AHI) of 30.0/h, more pronounced during REM sleep (40.2/h) and in the supine position (94.8/h). The examination also revealed significant sleep fragmentation (191 micro-awakenings; index of 27.4/h) and severe snoring (present in 64.6% of total sleep time), although with preserved overall sleep efficiency (95.3%). No relevant arrhythmias or significant periodic movements of the lower limbs were detected. A total of 209 respiratory events were observed (195 hypopneas, 12 obstructive apneas, and two central apneas), with a maximum duration of 27.3 s for apneas and 116.3 s for hypopneas. The oxygen desaturation index was 15.8 events per hour in the supine position and two events per hour in the non-supine position, with a minimum oxygen saturation of 92%. Spirometry demonstrated normal respiratory function for the parameters analyzed, with mildly reduced alveolar-capillary carbon monoxide transfer capacity; pulse oximetry showed normoxemia. The requested pulmonary computed tomography scan and echocardiogram showed normal results.

The FD referred the patient to pulmonology to start CPAP treatment, a therapy that significantly reduces snoring by stabilizing upper airway patency throughout sleep. The patient was also observed by an ENT specialist, but uvular surgery was not indicated because no focal palatal obstruction was identified on examination.

Currently, the patient reports improvement in symptoms with the initiation of CPAP use, reduced daytime sleepiness (ESS = 1 point), no need for naps, and a reduction in coffee consumption from six to two per day. There has been good adaptation to and adherence with the CPAP device, according to the patient himself and as evidenced by the equipment report. The patient reports that he takes the CPAP with him wherever he goes.

## Discussion

This case illustrates a severe form of OSAS confirmed on polysomnography by an AHI of 30 events/h, manifesting atypically in a young patient whose primary complaint was long-standing fatigue [21]. Ascertaining normal hemoglobin levels was essential, given hemoglobin's central role in oxygen transport, to exclude anemia as a contributor to the patient’s symptoms. Although anemia was excluded, mild iron deficiency was identified, providing a rationale for trial iron supplementation. Standard counseling included advising the patient to take iron on an empty stomach, to avoid concurrent intake of dairy products or other calcium-containing foods, and to consider vitamin C to improve absorption. Expected effects, such as gastrointestinal discomfort and darkened stools, were explained.

This young patient had severe snoring and markedly fragmented sleep, which plausibly accounted for the reported fatigue, daytime sleepiness, impaired concentration, perceived memory loss, with reduced quality of life and academic impact [21-23]. Intermittent nocturnal hypoxemia secondary to recurrent apneic events is a well-recognized pathophysiological mechanism underlying these manifestations. Furthermore, significant sleep fragmentation, evidenced by a high burden of microarousals (191 events), is consistent with the disease process and is known to adversely affect cognitive performance while increasing long-term cardiovascular risk. Despite multiple surgeries in childhood to improve nasal breathing, the patient has severe OSAS. These surgeries addressed pediatric nasal obstruction but did not prevent the later development of adult OSAS, especially without specific structural obstruction.

Digital clubbing is often associated with pulmonary or cardiovascular diseases such as lung cancer, interstitial pulmonary fibrosis, congestive heart failure, or infectious endocarditis. Less frequently, digital clubbing may occur in patients with extrathoracic diseases, including inflammatory bowel disease, liver cirrhosis, and gastrointestinal cancer. In this case, the physical examination, laboratory results, regular respiratory function tests, chest imaging, and echocardiogram, all without abnormalities, make other pulmonary or extrapulmonary causes of digital clubbing unlikely (Table 2).

**Table 2:** Digital clubbing differential workup. DC: digital clubbing; IBD: inflammatory bowel disease; LFTs: liver function tests

DC etiologies	Conditions considered	Tests performed	Findings	Rationale for exclusion
Pulmonary	Interstitial lung disease, lung cancer	Chest CT, spirometry	Normal	No evidence of parenchymal or neoplastic disease
Cardiac	Congenital cyanotic heart disease, heart failure, and endocarditis	Echocardiogram	Normal structure and function	No cardiac cause of hypoxia or clubbing
GI/hepatic	Cirrhosis, IBD	LFTs, clinical history	Normal	No hepatic or GI disease identified
Other	Thyroid disease, hematologic malignancy	Routine labs	Normal	Secondary systemic causes are unlikely

Digital clubbing is hypothesized to result from distal release of platelet-derived growth factors by megakaryocytes and platelet aggregates that escape pulmonary filtration, leading to vascular and connective tissue proliferation [24]. Its development appears to depend on chronic or repetitive hypoxemic exposure and disease-specific biology rather than a single oxygen saturation threshold. Although no defined SpO₂ cutoff predicts clubbing in intermittent hypoxia (e.g., OSAS), recurrent hypoxemia activates hypoxia-responsive pathways, including VEGF-mediated angiogenesis and platelet activation [25,26]. These mechanisms offer a biologically plausible explanation for clubbing in severe untreated OSAS, though direct clinical evidence remains limited [27].

Based on the severity of OSAS, CPAP therapy is clearly indicated and a priority. Early adaptation is essential for clinical improvement and the reduction of cardiovascular and metabolic risks. Other strategies, such as positional therapy, intraoral devices, or multilevel surgery, may be considered in cases of CPAP intolerance [21,23].

Adapting to CPAP can be challenging in young, physically active patients. Its use is often associated with obese and/or elderly individuals, which can affect self-esteem and even intimate relationships (in terms of the partner's perception and difficulties in adapting to the device). However, when there is an objective improvement in sleep quality and associated symptoms, this may be enough to overcome these limitations, as was the case here [7].

## Conclusions

This clinical case reinforces the importance of the role of the FD in the early identification of OSAS, even in young individuals without obvious comorbidities or classic risk factors. An atypical clinical picture is described in which longitudinal follow-up allowed for the assessment of persistent symptoms or signs, such as daytime sleepiness and difficulty concentrating.

It is crucial to emphasize that the management of OSAS is not limited to the use of CPAP. Successful intervention requires an emphasis on patient education, treatment adherence, and monitoring of clinical effects. In this context, active surveillance and early intervention by family doctors can prevent long periods of underdiagnosis, contributing to a significant improvement in patients' quality of life and reducing the risk of long-term complications.
